# Pathogenic Germline *BRCA1/2* Mutations and Familial Predisposition to Gastric Cancer

**DOI:** 10.1200/PO.18.00097

**Published:** 2018-07-05

**Authors:** Hiroshi Ichikawa, Toshifumi Wakai, Masayuki Nagahashi, Yoshifumi Shimada, Takaaki Hanyu, Yosuke Kano, Yusuke Muneoka, Takashi Ishikawa, Kazuyasu Takizawa, Yosuke Tajima, Jun Sakata, Takashi Kobayashi, Hitoshi Kemeyama, Hiroshi Yabusaki, Satoru Nakagawa, Nobuaki Sato, Takashi Kawasaki, Keiichi Homma, Shujiro Okuda, Stephen Lyle, Kazuaki Takabe

**Affiliations:** **Hiroshi Ichikawa**, **Toshifumi Wakai**, **Masayuki Nagahashi**, **Yoshifumi Shimada**, **Takaaki Hanyu**, **Yosuke Kano**, **Yosoke Tajima**, **Yusuke Muneoka**, **Takashi Ishikawa**, **Kazuyasu Takizawa**, **Jun Sakata**, **Takashi Kobayashi**, **Hitoshi Kemeyama**, and **Shujiro Okuda**, Niigata University Graduate School of Medical and Dental Sciences; **Hiroshi Yabusaki**, **Satoru Nakagawa**, **Nobuaki Sato**, **Takashi Kawasaki**, **Keiichi Homma**, Niigata Cancer Center Hospital, Niigata City, Niigata, Japan; **Stephen Lyle**, University of Massachusetts Medical School, Worcester, MA; and **Kazuaki Takabe**, Roswell Park Cancer Institute, and University at Buffalo, the State University of New York, Buffalo, NY.

## INTRODUCTION

Most gastric cancers (GCs) are considered sporadic; however, familial aggregation occurs in 10% of cases.^1,2^ Approximately 5% of GCs are caused by an autosomal dominant inherited trait, with carriers having a strongly increased risk of GC and other cancers.^3^ Clinical criteria for this entity were defined by the International Gastric Cancer Linkage Consortium.^4^ Among these, hereditary diffuse gastric cancer (HDGC) is a well-known type of familial GC (FGC). Approximately 40% of families fulfilling the clinical criteria for HDGC have germline *CDH1* mutations.^5^ A subset of the remaining families of HDGC, and ones fulfilling the criteria of other familial GC, harbor pathogenic germline mutation in other genes that are associated with hereditary cancer predisposition syndromes.^4^

Hereditary breast and ovarian cancer is one of the best-described inherited cancer predisposition syndromes, caused by pathogenic germline *BRCA1* or *BRCA2* (*BRCA1/2*) mutations.^6-9^ The increased risks of cancers other than breast and ovarian cancers were observed in the carriers.^10^ The association between germline *BRCA1/2* mutation and increased risk of GC were demonstrated in previous studies for families with hereditary breast and ovarian cancer.^11-14^ Regarding FGC, a recent large-scale study demonstrated that germline *BRCA2* mutations were identified in patients who had a family history that fulfilled the criteria of HDGC but were lacking *CDH1* mutations.^15^ Therefore, it is possible that germline *BRCA1/2* mutations may cause familial predisposition to GC.

Recent advances of comprehensive genomic analysis enable us to identify the genomic alterations in GC.^16^ *BRCA1/2* mutations were shown in the subset of GC tumor tissues; however, the association between germline *BRCA1/2* mutations and familial predisposition to GC were not fully understood. Previously, we performed genomic sequencing of 207 Japanese GCs using a 435-gene panel and identified *BRCA1/2* mutations in tumor.^17^ In this study, we conducted *BRCA1/2* genetic testing in seven Japanese patients with GC whose tumor had *BRCA1/2* mutations. We identified pathogenic germline *BRCA1/2* mutations in three patients who have a familial component of GC.

## METHODS

Among 28 patients whose tumor had *BRCA1/2* mutations in our previous study, seven patients who could provide written informed consent were enrolled. *BRCA1/2* genetic testing was performed by Myriad Genetic Laboratories using genomic DNA extracted from peripheral blood samples. We assessed the family history using the criteria for referral for genetic risk assessment and FGC defined by International Gastric Cancer Linkage Consortium after the enrollment (Table 1).^4^ Clinicopathological data are listed in Table 2. This study was approved by the institutional review boards at Niigata University and Niigata Cancer Center Hospital.

**Table 1.**
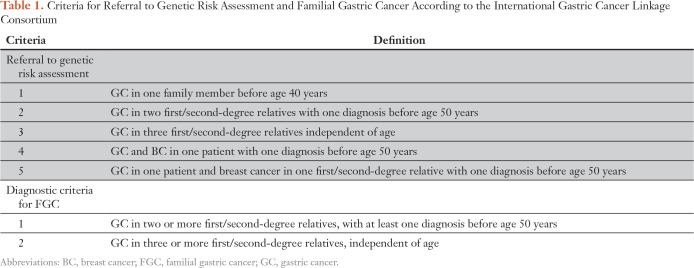
Criteria for Referral to Genetic Risk Assessment and Familial Gastric Cancer According to the International Gastric Cancer Linkage Consortium

**Table 2.**
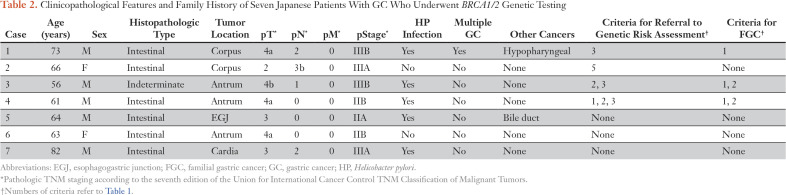
Clinicopathological Features and Family History of Seven Japanese Patients With GC Who Underwent *BRCA1/2* Genetic Testing

## RESULTS

Family history of cases 1 to 4 met the criteria for referral to genetic risk assessment, and that of cases 1, 3, and 4 met the criteria for FGC (Table 2). Pathogenic germline *BRCA1* and/or *BRCA2* mutations were detected in cases 1 to 3. Cases 5 to 7 also had germline *BRCA1* or *BRCA2* mutations; however, pathogenicity was determined to be uncertain. Case 4 had no germline mutations in *BRCA1/2* (Table 3). Somatic *BRCA1/2* mutations detected in tumor by using a 435-gene panel are detailed in Table 3. Germline *BRCA1/2* mutations in cases 1 to 3 were consistently detected in tumors.

**Table 3.**
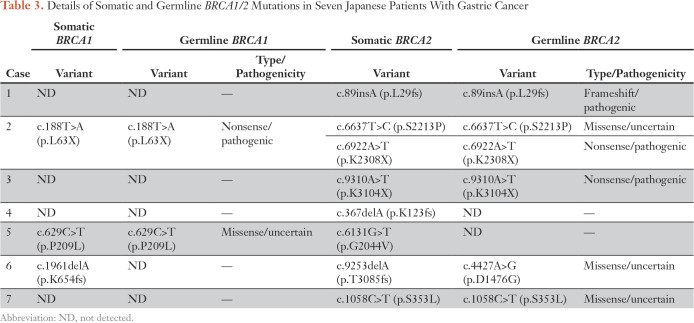
Details of Somatic and Germline *BRCA1/2* Mutations in Seven Japanese Patients With Gastric Cancer

### Case 1

**Fig 1.**
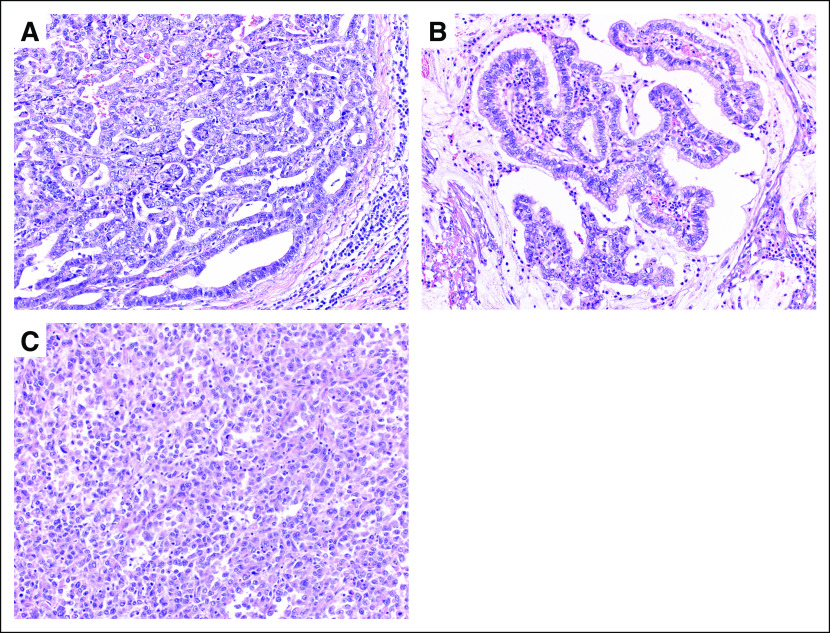
Histopathologic type of gastric cancer developed in three Japanese patients with pathogenic germline *BRCA1/2* mutations (hematoxylin and eosin, ×200). (A) Case 1: Moderately differentiated tubular adenocarcinoma of Lauren intestinal type. (B) Case 2: mucinous and moderately differentiated adenocarcinoma of Lauren intestinal type. (C) Case 3: poorly differentiated variant of tubular adenocarcinoma of Lauren indeterminate type.

**Fig 2.**
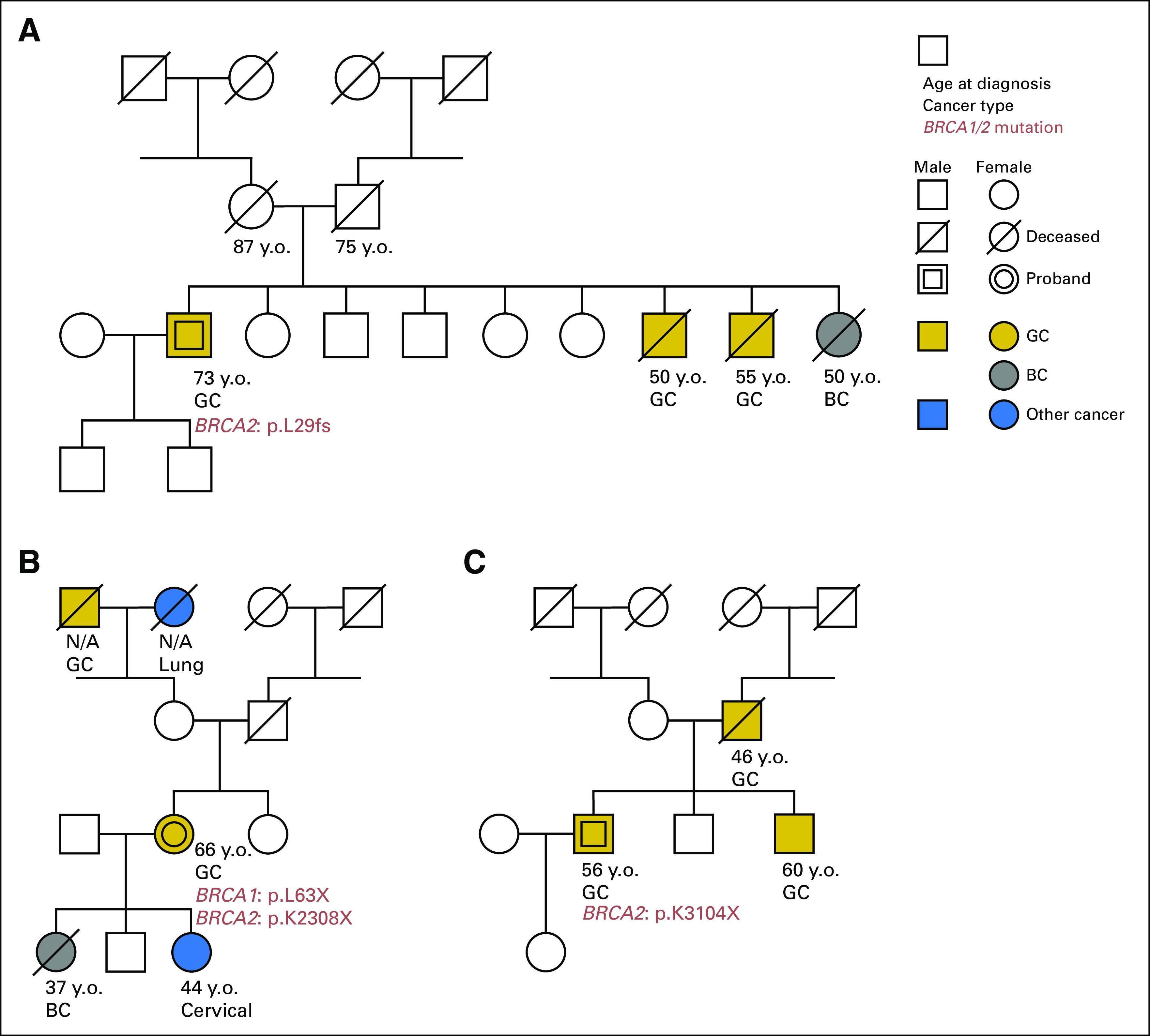
Family pedigrees of three Japanese patients with gastric cancer (GC) with pathogenic germline *BRCA1/2* mutations. (A) Case 1. (B) Case 2. (C) Case 3. Age in years (y.o.) is age at diagnosis. Gold, gray, and blue indicate relatives with GC, breast cancer (BC), and other cancers, respectively. N/A, not available.

A 73-year-old man had undergone endoscopic submucosal dissection for multiple intramucosal Lauren intestinal-type GCs on two occasions. Two years later, advanced GC and hypopharyngeal cancer were synchronously detected by periodic endoscopy. He underwent curative gastrectomy with D2 lymphadenectomy for GC. Moderately differentiated tubular adenocarcinoma classified as Lauren intestinal type was shown with pathologic T4aN2M0 (Fig 1A). *Helicobacter pylori* (HP) was identified in non-neoplastic mucosa by reviewing the hematoxylin and eosin–stained sections (Table 2). After that, he underwent concurrent chemoradiotherapy using cisplatin for hypopharyngeal cancer and achieved a complete response. He is alive with no evidence of recurrence 2 years after gastrectomy. He had a strong family history of cancers, with three GC and one breast cancer in first- or second-degree relatives (Fig 2A), which met the criteria for FGC (Table 1). Genetic testing detected a pathogenic frameshift germline mutation in *BRCA2* c.89insA (p.L29fs), which was identical to the variant detected in tumor (Table 3).

### Case 2

A 66-year-old woman underwent curative gastrectomy with D2 lymphadenectomy for GC. Mucinous and moderately differentiated adenocarcinoma classified as Lauren intestinal type was shown with pathologic T2N3bM0 (Fig 1B). HP was not identified in non-neoplastic mucosa (Table 2). Although adjuvant chemotherapy with S-1, an oral fluoropyrimidine preparation combining tegafur, gimeracil, and oteracil potassium, was performed, she suffered para-aortic lymph node recurrence 6 months after gastrectomy. Cisplatin with fluorouracil and paclitaxel was administered, and she achieved partial response. After 2 years of treatment, mediastinal lymph node metastasis subsequently developed, and chemotherapy was converted to cisplatin with CPT-11. All of the metastatic sites were markedly reduced and were maintained in reduced state for 7 months. Although there was a recurrence in the mediastinal lymph node, she is alive and continuing chemotherapy with paclitaxel and ramucirumab 5 years after postoperative recurrence. She had a strong family history of cancers, with two GCs and one case each of breast, lung, and cervical cancers in first- or second-degree relatives, which did not fulfill the criteria for FGC (Fig 2B). Genetic testing detected double pathogenic nonsense germline mutations in *BRCA1* c.188T>A (p.L63X) and in *BRCA2* c.6922A>T (p.K2308X), which were identical to the variants detected in tumor (Table 3).

### Case 3

A 56-year-old man underwent curative gastrectomy with D2 lymphadenectomy for GC. Poorly differentiated variant of tubular adenocarcinoma classified as Lauren indeterminate type was shown with pathologic T4bN1M0 (Fig 1C). HP was identified in non-neoplastic mucosa (Table 2). Adjuvant chemotherapy was performed with S-1 for 1 year, and he is alive with no evidence of recurrence 2 years after gastrectomy. He had a strong family history of GC, with three cases in first- or second-degree relatives, which met the criteria for FGC (Fig 2C). Genetic testing detected a pathogenic nonsense germline mutation in *BRCA2* c.9310A>T (p.K3104X), which was identical to the variant detected in tumor (Table 3).

## DISCUSSION

Previous studies have shown that germline *BRCA1/2* mutations increase the risk of GC.^11-14^ However, there is a paucity of data regarding the relationship between germline *BRCA1/2* mutations and familial predisposition to GC in Japan. In this report, we identified pathogenic germline *BRCA1/2* mutations in three Japanese patients with GC with a familial component of GC. Among these, one patient (case 2) had double pathogenic germline mutations in *BRCA1* and *BRCA2*, which were considered to be rare, as shown by a previous study for Ashkenazi Jewish double-founder mutations.^18^ To the best of our knowledge, this is the first report of Japanese patients with FGC harboring *BRCA1/2* mutations.

Multiple genetic and environmental factors influence the etiology of GC. The well-known environmental factors are high salt intake and HP infection, which cause chronic gastritis.^19^ The development of GC stands out in the family history of our three cases, rather than breast and ovarian cancers (Fig 2). HP infection was detected in cases 1 and 3 and intestinal-type GC, which is most associated with chronic gastritis, was observed in cases 1 and 2 (Fig 1). These findings suggested that chronic gastritis, which is associated with HP infection and/or environmental factors, may cause the loss of *BRCA1/2* function by the second-hit mutations in patients with the germline mutations. This synergistic exacerbatory effect confers the familial predisposition to GC in Japan, where a high incidence is observed.

*BRCA1/2* mutation carriers might have a risk of multiple GCs. In this report, case 1, with a *BRCA2* mutation, developed multiple metachronous GCs. *BRCA1/2* mutations are considered risk factors for ipsilateral breast cancer recurrence.^20^ Regarding HDGC caused by *CDH1* germline mutation, multiple foci of signet ring cell carcinoma were detected in the stomach of individual patients.^21,22^ Prophylactic total gastrectomy is recommended for *CDH1* mutation carriers because of the high cumulative incidence and the difficulty of early detection of HDGC by endoscopy.^23^ In this report, the intestinal type of GC, which is relatively easy to detect at an early stage by endoscopy, predominantly developed in the affected patients. Therefore, meticulous endoscopic surveillance may be recommended for *BRCA1/2* mutation carriers in Asia if additional large-scale studies substantiate the findings in our limited study.

*BRCA1/2* mutations have clinical implications in cancer treatment. *BRCA1/2*-mutated ovarian and breast cancers have high sensitivity to platinum chemotherapy and poly(adenosine diphosphate-ribose) polymerase (PARP) inhibitors because of defective DNA double-strand break repair.^24-26^ The US Food and Drug Administration has approved the PARP inhibitor olaparib for *BRCA1/2*-mutated advanced ovarian cancer. In this report, case 2, with both *BRCA1* and *BRCA2* germline mutations, showed high response of platinum-based chemotherapy for lymph node recurrence and long-term survival after the recurrence, in a manner similar to *BRCA1/2*-mutated ovarian and breast cancers. *BRCA1/2* genetic testing in GC might provide clinically useful information for the selection of therapeutic agents. Additional clinical studies are required to clarify whether the *BRCA1/2* mutations contribute to the good response to platinum-based chemotherapy and PARP inhibitors in GC.

In conclusion, *BRCA1/2* mutations may predispose to familial GC. *BRCA1/2* genetic testing in patients with GC with a familial component may help to optimize medical care, including cancer surveillance and the selection of treatment modalities in the era of precision medicine.
